# Assessment of knowledge, attitudes and practices of HIV post exposure prophylaxis among the doctors and nurses in Princess Marina Hospital, Gaborone: a cross-sectional study

**DOI:** 10.11604/pamj.2018.30.233.10556

**Published:** 2018-07-27

**Authors:** Peter Bareki, Tenego Tenego

**Affiliations:** 1 University of Botswana, School of Medicine and Princess Marina Hospital, Botswana

**Keywords:** Post exposure prophylaxis, health care workers, HIV- Human immunodeficiency virus

## Abstract

**Introduction:**

Botswana is one of the HIV/AIDS hardest hit countries in Sub-Saharan Africa with a prevalence of 17.6 percent while incidence is estimated to be 2.9 percent. The average risk of HIV transmission after a percutaneous exposure to HIV-infected blood has been estimated to be approximately 0.3% posing a threat to health care workers. This has resulted in HIV post exposure prophylaxis (PEP) being very important in the healthcare setting. The aim of this study was to assess knowledge, attitudes and practices of health care workers towards HIV PEP.

**Methods:**

A cross-sectional study was conducted at Princes Marina Hospital (PMH) in Gaborone from the 26th March-2nd April 2014. Inclusion criteria- registered medical doctors and nurses. Collected sample size was 199. Data was collected using self-administered questionnaires.

**Results:**

The majority of respondents 70.7% of the respondents had adequate knowledge about PEP, with 191(97.4%) of the study participants being aware of HIV PEP while 82.2% of the respondents had a positive attitude toward PEP. A significant number had been exposed 107(53.7%) to risky exposures. Of the exposed, 80(74.8%) took PEP, while 27(25.2%) did not take PEP. From the respondents that took PEP 21(26.6%) did not complete PEP, with 15(71.4%) quitting because of adverse side effects, 1(4.76%) assuming it was enough treatment and 1(4.76%) doubting drug efficacy.

**Conclusion:**

The participants were knowledgeable of the existence of HIV PEP and had a positive attitude toward the HIV PEP program. Although the participants were knowledgeable, they showed inadequate practices with regard to HIV PEP.

## Introduction

HIV/AIDS is a serious public health problem costing the lives of many people including health care workers. By the end of the year 2002, the world health organization estimated that 42 million people had been infected with the Human Immunodeficiency Viruses. In that year alone, 5 million new infections occurred with 75% of these new infections occurring in sub-Saharan Africa [1]. Therefore HIV/AIDS is probably the most serious disease and causes the highest level of anxiety amongst health care workers (HCWs) in many countries including in Botswana. Health care workers (HCWs) are persons working in health care setting and they are potentially exposed to infectious materials such as blood, tissue, specific body fluids, medical supplies, equipment or environmental surfaces contaminated with these substances [2]. They are frequently exposed to occupational hazards through percutaneous injury such as needle stick or cut with sharps, contact with the mucus membrane of eyes or mouth of an infected person, contact with non-intact skin exposed with blood or other potentially infectious body fluid. The WHO also estimates that overall, 90% of needle-stick injuries occur in low and middle-income countries [3]. There are recognized factors which have been associated with increased risk to acquiring HIV post occupational injury. In prospective studies of HCWs, the average risk of HIV transmission after a percutaneous exposure to HIV-infected blood has been estimated to be approximately 0.3% and after a mucous membrane exposure approximately 0.09% [4]. Injury with a hollow-bore needle is the commonest mode of infections. Other risk factors include depth of injury, visible contamination with the source patient’s blood, a procedure involving a needle placed directly in the source patient´s vein or artery and exposure to a source patient who died of acquired immunodeficiency syndrome [5]. Although episode of HIV transmission after non-intact skin has been documented, the average risk for transmission by this route has not been precisely quantified but estimated to be less than the risk for mucous membrane exposures [1].

There have been also findings that healthcare workers in Sub-Saharan Africa are at increased risk of infections from blood-borne pathogens because of the high prevalence of the pathogens and increased risk of occupational injuries. Unsafe practices like careless handling of contaminated needles, unnecessary injection on demand, re-use of inadequately sterilized needles and improper disposal of clinical waste increases risk for occupational exposure to blood-borne pathogens [6]. In light of increasing numbers of occupational needle stick injury among the health care workers across the world, World Health Organization has recommended that HIV post exposure prophylaxis should be provided to the affected population. Studies have shown that when administered shortly following exposure, PEP treatment reduces the risk of HIV infection by 81% [7]. WHO recommends that all health care institutions should have an easily accessible system in place available 24 hours a day that allows for reporting and managing the health care worker who experiences an occupational exposure to HIV. In the case of a needle stick injury, the area should be washed with soap and water. The wound should not be squeezed or milked or exposed to caustic agents such as bleach. For a cutaneous exposure, the area should be treated similarly with soap and water [8]. Since HIV PEP is not 100% effective, WHO/ILO has recommended that occupational safety rules be prioritized to minimize the accident predisposing healthcare worker to HIV [2]. With these recognized recommendations from the WHO, the implementation of the program has had many challenges especially in resource limited sub Saharan Africa where HIV/AIDS is more rampant [7]. Though there is irrefutable evidence supporting the effectiveness of the use of post exposure prophylaxis, African countries still have challenges to implementing the program because of various reasons which include poor resource management of limited resources, improper dissemination of the information about the program, poor program structures in many African countries [2]. In Malawi researchers have found that though the program is well implemented there are health care worker attitudes towards the program which affects their enrollment post exposure, which include fear of stigmatization and adverse side effect of the treatment. They also discovered that major shortcomings were insufficient awareness of the program among HCWs and poor follow up after the first consultation for PEP [6]. Similar results we also replicated in Ethiopia where they found lack of knowledge about PEP and fear of the process as the main factor affecting the enrollment among the HCW. Also literatures evidenced that there is an information gap in the health care setups [2]. For instance a study done in Guy’s and St Thomas’s hospital in London in 2001 indicated 93% of junior doctors had heard of PEP but fewer were aware that it reduced the rate of HIV transmission [6]. A national study in Kenya also showed, among those who were knowledgeable, only 45% sought HIV PEP. The main reasons for not seeking PEP among this group was lack of sufficient information (35%) followed by fear of the process and what could follow (28%) [9].

In Botswana, where the prevalence of HIV/AIDS is high, health care workers face the similar challenges faced by HCW elsewhere [10]. This has actuated the implementation of the PEP program for occupational purpose [11]. Though the program has been implemented for more than 10 years now, there is no published research about the awareness, attitude and use of the PEP among the healthcare workers in Botswana. Neither is there any documented review of the project. Though there is a clear guide for PEP in the Botswana treatment guideline there is no clear outline on how the program should be implemented [10]. This research assessed the knowledge, attitudes and practices of post exposure prophylaxis among the healthcare workers in Princess Marina hospital, Gaborone/Botswana. The research was of higher importance and also appropriate in our setting where we have a very high prevalence of HIV/AIDS. The results will help the policy makers and the managers on how they could better improve the PEP program. We conducted this study to assess knowledge, attitude and practice of post exposure prophylaxis among the healthcare workers in Princess Marina Hospital.

## Methods

### Operational definitions

*Post-exposure prophylaxis*- is an emergency medical response that can be used to protect individuals exposed to the human immunodeficiency virus (HIV). *Healthcare workers (HCWs)* in this study will include registered nurses and medical doctors. We chose nurses and doctors because of the limited duration in which the study will be conducted, and because they have direct contact with the patients.

### Study area

The research study will be conducted in Gaborone the capital city of the republic of Botswana. Located in south east region of the country, Gaborone has an estimated population of about 231,626, with an estimated 17,773 Gaborone citizens, 17.1% of the total population of Gaborone, have tested positive for HIV. Princess Marina Hospital was established in 1966 and became operational in 1967 doubling as maternity clinic. It is the main referral hospital in Botswana (Gaborone) and is currently housing 500 beds is located in Gaborone. Gaborone is also considered one of the fastest growing cities in Africa.

### Study design

The study was a cross-sectional study based on health care workers in Princess Marina Hospital the largest referral hospital in Botswana. We chose a cross sectional study to assess the knowledge, attitudes and practices of HIV PEP amongst HCW in PMH with respect to presence and absence of exposure to HIV contaminated body fluids/equipment. The other reason for our choice is that cross sectional studies are relatively quick to carry out, looking at the limited time we have to carry out the study and limited resources.

**Source population:** The source population was the current health care workers in Gaborone.

### Study population

The inclusion criteria used for selection of the study population from the source population was as follows: Being a registered nurse at PMH; Being a registered medical doctor at PMH. Exclusion criteria was: all porters and cleaners; being a nursing student or medical student in clinical attachment at PMH; a registered laboratory technicians. We decided to include nurses and doctors because of the limited duration in which the study will be conducted, and because they have direct contact with the patients and are exposed to HIV positive patients blood while carrying out procedures.

### Sample size

The study includes medical officers and nurses at Princess Marina Hospital who are 101 and 593 respectively. Thus the total of the study population will be 693. Sample size was calculated using Stat calc within the EPI info application. At 95% CI and the expected frequency of the knowledge about PEP of 50%, with the worst acceptable result set within the limit 45-55% from the sample size was found to be 247.

### Sampling procedure

A non-probabilistic sampling (Availability sampling) method was used to enroll the subjects. The researchers distributed the questionnaires to available and consenting individuals. Informed consent in written form (in English or Setswana based on the participant’s preference) were obtained from all study participants before proceeding with data collection. Respondents were then be given questionnaires for self-administration. Questionnaires were dropped into a ballot box in-front of the participants to assure them that privacy was maintained.

### Scoring of knowledge, attitudes and practices

Four questions from the questionnaire were used to assess the knowledge of respondents about PEP for HIV and those who scored greater than or equal to 70% were considered knowledgeable. Fours questions (1-4) from Table 1 were used to assess participants’ attitude towards PEP for HIV and those who scored 70% and above were considered as having good attitude. Correct answers to the direct knowledge questions 4, 5, 6, 7, were averaged from Table 2. Practices were assessed by comparison with other studies.

**Table 1:** Sociodemographic characteristics of HCW’s in Princess Marina Hospital/Gaborone-Botswana, 2014

Question	Response	Frequency	Percentage
Age of respondents	**20-30**	**85/197**	**43.1%**
	31-40	70/197	35.5%
	**41-50**	**32/197**	**16.2%**
	Over 50	10/197	5.1%
Sex	**Male**	**84/196**	**42.9%**
	Female	112/196	57.1%
Work experience	**6 months-2 years**	**50/197**	**25.4%**
	3-5 years	47/197	23.9%
	**6-8 years**	**36/197**	**18.3%**
	Over 8 years	64/197	32.5%
Marital status	**Married**	**89/197**	**45.2%**
	Single	102/197	51.8%
	**Divorced**	**5/197**	**2.5%**
	Widowed	1/197	0.5%
Religion	**Christian**	**157/167**	**94.0%**
	Buddhism	1/167	0.6%
	**Muslim**	**4/167**	**2.4%**
	Hindu	3/167	1.8%
	**Atheist**	**1/167**	**0.6%**
	Sikh	1/167	0.6%
Profession	**Medical doctor**	**78/197**	**39.6%**
	Nurse	105/197	53.3%
	**Midwife**	**13/197**	**6.6%**
	Other	1/197	0.5%
Educational level	**Certificate**	**1/198**	**0.5%**
	Diploma	77/198	38.9%
	**First degree**	**101/198**	**51.0%**
	Master’s degree	11/198	5.6%
	**Specialist**	**8/198**	**4.0%**

**Table 2:** Response of HCWs to each question that assess their knowledge about PEP in Princess Marina Hospital/Gaborone-Botswana, 2014

Questions	Response	Frequency	Percentage
1.Heard about PEP	**Yes**	**191/196**	**97.4%**
	NO	5/196	2.6%
2. From what source you got the information?	**Training**	**113/199**	**56.8%**
	Mass media	17/199	8.5%
	**Friends**	**31/199**	**15.6%**
	Journals	37/199	18.6%
3. When do you think PEP should be given?	**When the source patient is high risk for HIV.**	**56/199**	**28.1%**
	When the patient is known to be HIV positive.	49/199	24.6%
	**When the HIV status of the source patient is unknown.**	**39/199**	**19.6%**
	For any needle stick injury in the work place.	123/199	61.8%
4. What is the maximum delay to take PEP?	**12hours**	**27/198**	**13.6%**
	24hours	41/198	20.7%
	**48hours**	**22/198**	**11.1%**
	72hours	108/198	54.5%
5. What is the preferable time to take PEP?	**Within 1 hour of exposure.**	**90/194**	**46.4%**
	Within 6 hours of exposure.	71/194	36.6%
	**Within 12 hours of exposure.**	**24/194**	**12.4%**
	Within 72 hours of exposure.	9/194	4.6%
5. What is the effectiveness of PEP?	**100%**	**17/193**	**8.8%**
	80-100%	163/193	84.5%
	**60-70%**	**12/193**	**6.2%**
	30-50%	1/193	0.5%
6. What is the length of time to take PEP?	**For 28 days**	**187/192**	**97.4%**
	For 40 days	3/192	1.6%
	**For 6 months**	**2/192**	**1.0%**
7. Have you ever attended any training for PEP?	Yes	79/196	40.3%
	**No**	**117/196**	**59.7%**

### Ethical approval

The ethical committees that approved the study to be conducted were the University of Botswana Institutional Review Board, (Botswana) Ministry of Health Ethics Committee and the Princess Marina Ethics Committee. All participants took part in the study after informed consent was obtained from the subjects.

## Results

### Sociodemographic characteristics

Although our calculated sample size was 247, we only managed to get 199 respondents. From the 199 respondents that answered and returned the questionnaires, a total of 84(42.9%) males and 122(57.1%) females responded in this study (with a total of 196 known gender respondents while 3 respondents did not respond to the question). Most respondents 85(43.1%) were aged 20-30 years, 70(35.5%) were aged 31-40 years, 32(16.2%) were aged 41-50 years. From the respondents 105(53.3%) were nurses, 78(39.6%) were medical doctors, 13(6.6) were midwives. The majority of respondents had a first degree 101(51.0%), followed by diploma77 (38.9%), and master’s degree 11(5.6%). With regard to the year of service of the respondents (HCW’s), 50(25.4%) served 0.5-2 years, 47(23.9%) served 3-5 years, 36(18.3%) served 6-8 years, and lastly 64(32.5%) served for over 8 years. This information is reflected in Table 1 below.

### Knowledge level of the HCWs about PEP for HIV

Knowledge was assessed using the questions represented in Table 2 below. The majority of health care workers in Princess Marina hospital 191(97.4%) have heard about PEP, with most of their knowledge having been obtained through formal training 113(56.8%). The study participants 163(84.5%) think that HIV PEP is effective. There was however a knowledge gap amongst study participants with regard to when to start PEP, with 90(46.4%) knowing when to initiate PEP. Only 108(54.5%) of the respondents knew the maximum delay time to take PEP and 187(97.4%) knew how long exposed HCW should be enrolled on PEP to prevent infection/seroconversion.

### Attitude of the HCWs about PEP for HIV

The majority of respondents 164(82.2%) had a positive attitude toward PEP. The study respondents 184(93.9%) agreed that HIV PEP is important, while 167(85.6%) believe that training of PEP is important for behavioral change amongst HCW’s towards PEP. When asked about the need for PEP in the work areas, many showed a positive attitude, with 186(94.9%) strongly agreeing to this suggestion. The majority 166(84.3%) of respondents believing that PEP reduces the likelihood of being HIV positive, with 75(38.5%) believing that HIV PEP prevents other infections(hepatitis B and C) while 104(53.3%) disagree with this notion. The belief that PEP is indicated for any type of sharp object injury was also assessed among the respondents and it was noted that 101(53.2%) agreed, while 89(46.8%) disagreed with this saying. This information is reflected in Table 3 below.

**Table 3:** Attitude of HCWs about PEP in Princess Marina Hospital, Gaborone-Botswana, 2014

Question	Response	Frequency	Percentage (%)
Do you think PEP is important?	Yes	184/196	93.87
	**No**	**6/196**	**3.06**
	I am not sure	6/196	3.06
Do you believe that training of PEP is important for a behavioral change?	**Agree**	**167/195**	**85.6**
	Disagree	19/195	9.7
	**Neutral**	**9/195**	**4.6**
Do you think there should be a PEP guideline in the work areas?	Strongly agree	186/196	94.9
	**Agree**	**9/196**	**4.6**
	Disagree	1/196	0.5
Do you believe PEP reduces the likelihood of being HIV positive?	**Yes**	**166/197**	**84.3**
	No	16/197	8.1
	**I’m not sure**	**15/197**	**7.6**
Do you believe HIV PEP to prevent other infections (Hepatitis B & C)?	Agree	75/195	38.5
	**Partially agree**	**16/195**	**8.2**
	Disagree	104/195	53.3
How do you see the saying that, “PEP is indicated for any type of sharps injuries”?	Agree	101/190	53.2
	**Disagree**	**89/190**	**46.8**
What is your opinion on the belief that PEP is not important if the exposure is not with blood of a known HIV positive patient?	Agree	26/194	13.4
	**Disagree**	**168/194**	**86.6**

### Practice status of the HCWs towards PEP for HIV

Among all of the respondents 107(53.7%) had been exposed to HIV risky conditions and of these exposed respondents, 80(74.8%) took PEP. On the other hand, 27(25.2%) of the exposed did not take PEP. From the respondents who took PEP, 50(62.5%) reasoned that they took PEP after being exposed to known HIV positive blood, 12(15%) were exposed to blood from a patient whose HIV status was unknown, 25(31.8%) took PEP because of injury from contaminated sharps, 8(10%) was because of contact with patient body fluids. Among the respondents that took PEP, only 3(3.8%) started PEP after the recommended initiation time. From the respondents that took PEP, only 57(72.2%) completed the PEP treatment regimen/period (28 days). From the respondents that took PEP 21(26.6%) did not complete PEP, with 15(71.4%) quitting because of adverse side effects, 1(4.76%) assuming it was enough treatment, 1(4.76%) doubting drug efficacy. The results are shown in Table 4 below.

**Table 4:** Practice of PEP for HIV among HCW in Princess Marina Hospital, Gaborone/Botswana, 2014

Question	Response	Frequency	Percentage (%)
Have you ever been exposed to HIV risky conditions (i.e. sharp object injuries, body fluid splashes) at the workplace?	Yes	107/199	53.7%
	**No**	**87/199**	**43.7%**
	I do not know	5/199	2.5%
Took PEP after exposure	**Yes**	**80/107**	**74.8%**
	No	27/107	25.2%
The reason the respondent took PEP	**Exposure to blood from known HIV positive patient**	**50/107**	**46.7%**
	Exposure to blood from patient whose HIV status is unknown	12/107	11.2%
	**Injury from any sharp objects**	**25/107**	**23.4%**
	Contact with patient body fluids	8/107	7.5%
Reasons for not taking HIV PEP	**Patient was HIV negative**	**2/107**	**1.9%**
	Because of adverse side effects of ARV’s	9/107	8.4%
The time you started taking PEP	**Within 1 hour of exposure**	**24/79**	**30.4%**
	Within 72 hours of exposure	3/79	8.8%
	**Within 2-6 hours of exposure**	**35/79**	**44.3%**
	Within 6-10 hours of exposure	17/79	21.5%
The period of time that you the respondent took PEP	**1-7 days**	**13/79**	**16.5%**
	8-14 days	9/79	11.4%
	**28 days**	**57/79**	**72.2%**
Completed the prescribed drugs for PEP	Yes	58/79	73.4%
	**No**	**21/79**	**26.6%**
What was your reason for discontinuation of PEP drugs	Fear of adverse side effects	15/21	71.4%
	**Assuming that it was enough**	**1/21**	**4.8%**

## Discussion

The strengths of this study were that knowledge, attitudes of health care workers in Princess Marina hospital have never been investigated, hence the study results will offer input into the enhancement to personal safety and PEP enrolment by healthcare workers. Another strength was the proximity of the hospital to the University of Botswana School of medicine, making it easy to quickly gain assistance from our supervisor and to travel to and from both facilities while conducting data collection. Limitations of the study were: the questionnaire was not pretested; Data collection commenced late due the lengthy process of study approvals by 3 different ethics clearance committees/IRB’s (University of Botswana, Ministry of Botswana, Princess Marina Hospital); Study was conduct during the working days when the target population was busy attending to patients, together with working in day and night shifts, requiring us to collect data at both daytime and night to capture more respondents; Some questionnaires were not returned.

### Knowledge

From the results obtained from the respondents, 191(97.4%) have heard about HIV PEP. These results are similar with a study conducted in University of Abuja Teaching hospital, Nigeria which had a majority 97% of respondents had heard about PEP [12]. In a study in Gondar University hospital, 2012, 181(92.8%) of the HCWs had heard about HIV post exposure prophylaxis [7]. In another study conducted on general practitioners in northern Sydney Australia, 68.5% were aware of the availability of HIV PEP for high risk occupational exposures [13], of which this study shows a higher awareness (97.4%). The reason for this lower level of awareness could be because of the lower prevalence of HIV and other blood borne diseases like hepatitis B and C in Australia as compared to sub-Saharan setting, like Botswana and Ethiopia, requiring healthcare workers to be knowledgeable about HIV PEP because of the increased risk of exposure. 90/194(46.4%) of the respondents who responded to the question on when to start PEP for HIV correctly chose, “within an hour of exposure”. Comparing this study with a similar study conducted amongst HCW’s in Gondar, North west Ethiopia with 50.8% of their respondents answering that PEP should be started within 1 hour [7]. This low percentage (46.4%), may be similar to the Ethiopian study because of geographic proximity and access to information, emigration of clinicians amongst countries. This study’s results shows that 70.7% of respondents had adequate knowledge as they scored more than 70%. (The 70.7% was obtaining after averaging questions 4, 5, 6, 7, which are direct knowledge questions, look at Table 2 below assessing knowledge).

**Attitudes:** The majority of respondents 164(82.2%) had a positive attitude toward PEP. This possibly being so because of the adequate level of knowledge shown by the respondents with regard to HIV PEP.

### Practices

A significant number of respondents 107(53.7%) had been exposed to blood, body fluids, sharp objects while caring for patients. Among the exposed, 80(74.8%) took PEP, while 31 (29%) did not take PEP. The percentage of respondents that were exposed to HIV risky conditions was less as compared to a similar study conducted in the Jimma zone of Southwest Ethiopia, in which 174 (68.9%) HCW had been exposed to HIV risky conditions, and out of the 174 exposed HCWs, 142 (81.6%) did not use post exposure prophylaxis [2]. The results of this study indicate that a higher percentage (72.1%) of the exposed HCWs was initiated on HIV PEP. This may be because of the fact that 213 (83.9%) of the Jimma zone respondents had inadequate knowledge about HIV PEP [2], as compared to this study’s respondents in which 97.4% of respondents were aware of PEP. Only 3 (3.8%) started taking PEP after the recommended initiation time. The failure of exposed respondents to enroll in PEP may be explained by the 29.3% of respondents who had inadequate knowledge about PEP. The results of this study indicate that the initiation and completion of HIV PEP is based on the knowledge of HCWs with regard to the subject matter. Amongst those who took PEP, 21 (26.3%) failed to complete PEP, while 58 (72.5%) completed the 28 day regimen. These findings were consistent with the Gondar study, in which 60.9% of respondents managed to complete the regimen.

## Conclusion

The participants were aware of the existence of HIV PEP, and were knowledgeable concerning the program. Although the participants were knowledgeable, they showed inadequate practices with regard to HIV PEP, hence this should be exploited, and treated as an opportunity to improve the practices of PEP among the HCWs. This can be done by providing formal training (universal safety procedures/standard precautions) for all health care workers and support structures like establishing a 24 hour accessible PEP center, with a clear guideline on those who fit PEP enrollment criteria, a guideline detailing the PEP regimen, so as to improve initiation and completion of the PEP regimen.

### What is known about this topic

- PEP is used in an acute (within 72 hours) setting after HIV exposure;
- A combination of Highly Active Antiretroviral Drugs (HAART) is used to prevent HIV seroconversion;
- PEP is only used on HIV negative persons that have been exposed; An HIV test is required before starting PEP.

### What this study adds

- Having a good knowledge base about HIV PEP does not guarantee starting or completing PEP by healthcare workers;
- Attitudes towards HIV (together with stigmas associated with HIV) and its exposure hinder health workers from starting PEP;
- Even with good knowledge and attitudes about HIV PEP, poor practices with regard to adhering to PEP have been exposed by healthcare workers in the study and this is where interventions should be focused.

## Competing interests

The authors declare no competing interest.
